# Supplementary material for: Polyketide synthase gene clusters in the ericoid mycorrhizal fungus Oidiodendron maius: genome-wide prediction, expression profiling, and functional characterization of knockout mutants
Source: Front Plant Sci. 2026 Sep 16;17:1924430. doi: 10.3389/fpls.2026.1924430 (PMC13623903; doi:10.3389/fpls.2026.1924430)
Supplement: Supplementary file 1 [file SupplementaryFile1.zip › Supplementary Materials/Supplementary Folder 1.zip!/Oidma1_AssemblyScaffolds_Repeatmasked/knownclusterblast/cluster53/jgi.p_Oidma1_106945_mibig_hits.html]

| MIBiG Protein | Description | MIBiG Cluster | MiBiG Product | % ID | % Coverage | BLAST Score | E-value |
| --- | --- | --- | --- | --- | --- | --- | --- |
| ACB12564.1 | Fum19 | BGC0000063 | Fumonisin\_biosynthetic\_gene\_cluster | 44 | 102.430790007 | 1132 | 0.0 |
| AAN74822.1 | Fum19p | BGC0000062 | Fumonisin\_biosynthetic\_gene\_cluster | 44 | 102.430790007 | 1131 | 0.0 |
| OAQ83754.1 | ABC\_multidrug\_transporter | BGC0001358 | Leucinostatins\_biosynthetic\_gene\_cluster | 36 | 106.819716408 | 804 | 2.2e-232 |
| AMM63163.1 | AniL | BGC0001371 | Echinocandin\_B\_biosynthetic\_gene\_cluster | 34 | 100.607697502 | 746 | 5.3e-215 |
| EPE34342.1 | P-loop\_containing\_nucleoside\_triphosphate\_hydrolase | BGC0001035 | Pneumocandin\_biosynthetic\_gene\_cluster | 36 | 91.1546252532 | 745 | 9.1e-215 |
| AFT91383.1 | EcdL | BGC0000338 | Echinocandin\_B\_biosynthetic\_gene\_cluster | 33 | 101.958136394 | 713 | 5e-205 |
| AMR44284.1 | ABC\_transporter | BGC0001398 | Phomopsins\_biosynthetic\_gene\_cluster | 29 | 87.9810938555 | 471 | 4.3e-132 |
| CRG85577.1 | hypothetical\_protein | BGC0001402 | Cyclochlorotine\_biosynthetic\_gene\_cluster | 25 | 102.160702228 | 309 | 1.7e-83 |
| AET14838.1 | multidrug\_resistance\_protein | BGC0001274\_c2 | Non-acetylated\_open-chain\_sophorolipid\_/\_diacetylated\_lactonic | 22 | 62.1877110061 | 129 | 2.4e-29 |
| CAG34715.1 | putative\_ABC\_transporter | BGC0000713 | Ribostamycin\_biosynthetic\_gene\_cluster | 27 | 35.786630655 | 125 | 5.8e-28 |
| CAH58693.1 | putative\_exporter | BGC0000711 | Neomycin\_biosynthetic\_gene\_cluster | 27 | 35.5165428764 | 123 | 2.2e-27 |
| CAF33315.1 | putative\_ABC\_transporter,\_ATP-binding\_protein,\_NeoU | BGC0000709 | Neomycin\_biosynthetic\_gene\_cluster | 27 | 35.5165428764 | 123 | 2.2e-27 |
| CAH05098.1 | ABC\_transport\_protein | BGC0000710 | Neomycin\_biosynthetic\_gene\_cluster | 27 | 35.5165428764 | 123 | 2.2e-27 |
| AAG28766.1 | CoaD | BGC0000617 | Coagulin\_biosynthetic\_gene\_cluster | 31 | 22.4848075625 | 116 | 2.7e-25 |
| BAE56595.1 |  | BGC0001123 | Aspirochlorine\_biosynthetic\_gene\_cluster | 26 | 30.5199189737 | 114 | 1e-24 |
| ALX37944.1 | GaeT | BGC0001388 | Gassericin\_E\_biosynthetic\_gene\_cluster | 26 | 24.9831195138 | 113 | 3e-24 |
| BAM09402.1 | putative\_ABC-transporter | BGC0000619 | Gassericin\_T\_biosynthetic\_gene\_cluster | 26 | 24.9831195138 | 112 | 3.9e-24 |
| EDY04298.1 | ABC\_transporter-related\_protein | BGC0000516 | Geobacillin\_II\_biosynthetic\_gene\_cluster | 25 | 35.5165428764 | 105 | 4.8e-22 |
| ABQ84449.1 | salivaricin\_ABC-transporter | BGC0000624 | Salivaricin\_CRL1328\_alpha\_peptide\_/\_salivaricin\_CRL1328\_beta\_peptide | 25 | 25.9959486833 | 102 | 5.3e-21 |
| BAJ19059.1 | putative\_ABC\_transporter | BGC0000288 | A-503083\_biosynthetic\_gene\_cluster | 28 | 31.2626603646 | 101 | 9e-21 |
| ANP43732.1 | ThsT | BGC0001391 | Thusin\_biosynthetic\_gene\_cluster | 28 | 20.8642808913 | 101 | 9e-21 |
| AAX31531.1 | putative\_ABC\_transporter | BGC0000336 | Daptomycin\_biosynthetic\_gene\_cluster | 25 | 37.4071573261 | 101 | 9e-21 |
| AFJ14797.1 | PlpH | BGC0000403 | Pelgipeptin\_biosynthetic\_gene\_cluster | 28 | 29.9797434166 | 101 | 1.2e-20 |
| AGM16416.1 | paenibacterin\_ABC-transporter\_E | BGC0000400 | Paenibacterin\_biosynthetic\_gene\_cluster | 28 | 20.9993247806 | 99 | 3.4e-20 |
| BAI23328.1 | putative\_ABC-transporter | BGC0000949 | A-500359s\_biosynthetic\_gene\_cluster | 28 | 31.5327481431 | 99 | 4.5e-20 |
| ADX66465.1 | ScnB | BGC0000108 | Natamycin\_biosynthetic\_gene\_cluster | 26 | 33.9635381499 | 98 | 5.8e-20 |
| AAK73498.1 | AmphG | BGC0000015 | Amphotericin\_biosynthetic\_gene\_cluster | 26 | 35.7191087103 | 97 | 1e-19 |
| AAZ76601.1 | BhtT | BGC0000497 | BhtA1\_/\_BhtA2\_biosynthetic\_gene\_cluster | 23 | 32.1404456448 | 97 | 1.3e-19 |
| ACQ63630.1 | ABC\_multidrug\_resistance\_transporter | BGC0000875 | Caprazamycin\_biosynthetic\_gene\_cluster | 24 | 36.1917623228 | 96 | 2.2e-19 |
| CAC20925.1 | PimB\_protein | BGC0000125 | Pimaricin\_biosynthetic\_gene\_cluster | 25 | 36.7319378798 | 96 | 2.9e-19 |
| ACA97578.1 | PmxC | BGC0000408 | Polymyxin\_biosynthetic\_gene\_cluster | 27 | 20.8642808913 | 96 | 3.8e-19 |
| EEM14530.1 | hypothetical\_protein | BGC0001210 | Pseudomycoicidin\_biosynthetic\_gene\_cluster | 26 | 23.8352464551 | 95 | 4.9e-19 |
| CAJ87587.1 | putative\_ABC\_transporter\_protein | BGC0001055 | Yersiniabactin\_biosynthetic\_gene\_cluster | 28 | 25.0506414585 | 95 | 6.5e-19 |
| ABP54659.1 | ABC\_transporter\_related | BGC0000241 | Lomaiviticin\_biosynthetic\_gene\_cluster | 25 | 37.2721134369 | 95 | 6.5e-19 |
| AHF21227.1 | TriC | BGC0000449 | Tridecaptin\_biosynthetic\_gene\_cluster | 25 | 29.5746117488 | 95 | 6.5e-19 |
| BAD72775.1 |  | BGC0000552 | Smb\_A\_/\_Smb\_B\_biosynthetic\_gene\_cluster | 23 | 32.6130992573 | 94 | 1.1e-18 |
| AHZ61877.1 | Lom43 | BGC0000240 | Lomaiviticin\_biosynthetic\_gene\_cluster | 24 | 37.2045914922 | 93 | 2.5e-18 |
| WP\_006126653.1 | ABC\_transporter | BGC0000554 | SRO15-3108\_biosynthetic\_gene\_cluster | 26 | 34.3686698177 | 92 | 3.2e-18 |
| AAF71763.1 | NysG | BGC0000115 | Nystatin\_biosynthetic\_gene\_cluster | 23 | 36.6644159352 | 92 | 4.2e-18 |
| WP\_010071619.1 | ABC\_transporter\_ATP-binding\_protein | BGC0000578 | SRO15-2005\_biosynthetic\_gene\_cluster | 24 | 37.2721134369 | 92 | 5.5e-18 |
| BAB04170.1 | lantibiotic\_mersacidin\_transporter\_system | BGC0000517 | Haloduracin\_alpha\_/\_haloduracin\_beta\_biosynthetic\_gene\_cluster | 24 | 34.0310600945 | 92 | 5.5e-18 |
| WP\_020635023.1 | multidrug\_ABC\_transporter\_permease | BGC0001211 | Albachelin\_biosynthetic\_gene\_cluster | 23 | 35.5840648211 | 91 | 9.3e-18 |
| AAK73499.1 | AmphH | BGC0000015 | Amphotericin\_biosynthetic\_gene\_cluster | 27 | 26.4010803511 | 91 | 9.3e-18 |
| XP\_011392729.1 | ABC\_transporter | BGC0001281 | Ustilagic\_acid\_biosynthetic\_gene\_cluster | 25 | 27.0762997974 | 91 | 1.2e-17 |
| CAD91219.1 | putative\_ABC\_transporter,\_Dbv24 | BGC0000289 | A40926\_biosynthetic\_gene\_cluster | 24 | 28.7643484132 | 88 | 6e-17 |
| CBW74823.1 | Multidrug\_resistance\_ABC\_transporter\_ATP-binding\_and\_permease\_protein | BGC0000571 | Burhizin\_biosynthetic\_gene\_cluster | 27 | 29.1019581364 | 87 | 1e-16 |
| EFI65097.1 | lantibiotic\_transport\_protein | BGC0001407 | Bicereucin\_biosynthetic\_gene\_cluster | 22 | 37.0020256583 | 86 | 3e-16 |
| BAJ05895.1 | putative\_ABC\_multidrug\_resistance\_transporter | BGC0000872 | A-90289\_biosynthetic\_gene\_cluster | 25 | 26.4010803511 | 86 | 3e-16 |
| CAG38699.1 | putative\_ABC-transporter,\_ATP-binding\_protein | BGC0000708 | Lividomycin\_biosynthetic\_gene\_cluster | 22 | 33.6259284267 | 86 | 3e-16 |
| AHF21240.1 | CrnT | BGC0000500 | Carnolysin\_A1\_/\_carnolysin\_A2\_biosynthetic\_gene\_cluster | 26 | 23.4976367319 | 84 | 1.1e-15 |
| ABV83218.1 | CppG | BGC0000116 | Nystatin-like\_Pseudonocardia\_polyene\_biosynthetic\_gene\_cluster | 26 | 36.8669817691 | 84 | 1.5e-15 |
| CAC20924.1 | PimA\_protein | BGC0000125 | Pimaricin\_biosynthetic\_gene\_cluster | 26 | 29.3045239703 | 80 | 2.1e-14 |
| AAM80540.1 | StaU | BGC0000290 | A47934\_biosynthetic\_gene\_cluster | 25 | 24.3079000675 | 80 | 2.1e-14 |
| ABD65953.1 | ABC\_transporter | BGC0000341 | Enduracidin\_biosynthetic\_gene\_cluster | 24 | 22.0796758947 | 78 | 6.3e-14 |
| AFS60105.1 | PaenT | BGC0000540 | Paenibacillin\_biosynthetic\_gene\_cluster | 25 | 23.0249831195 | 77 | 1.1e-13 |
| ABS50189.1 | putative\_ABC\_transporter\_permease/ATP-binding\_protein | BGC0000410 | Pseudomonine\_biosynthetic\_gene\_cluster | 22 | 35.5840648211 | 77 | 1.1e-13 |
| ADX66464.1 | ScnA | BGC0000108 | Natamycin\_biosynthetic\_gene\_cluster | 26 | 31.6677920324 | 77 | 1.4e-13 |
| AGL93176.1 | ABC\_transporter\_ATP-binding\_protein | BGC0000560 | Subtilomycin\_biosynthetic\_gene\_cluster | 25 | 25.7933828494 | 77 | 1.8e-13 |
| CAB01940.1 | ExpD1 | BGC0000801 | Galactoglucan\_biosynthetic\_gene\_cluster | 24 | 26.3335584065 | 76 | 4.1e-13 |
| ABV83219.1 | CppH | BGC0000116 | Nystatin-like\_Pseudonocardia\_polyene\_biosynthetic\_gene\_cluster | 27 | 24.4429439568 | 72 | 4.5e-12 |
| AAC72259.1 | LctT | BGC0000521 | Lacticin\_481\_biosynthetic\_gene\_cluster | 26 | 21.8771100608 | 71 | 7.6e-12 |
| AGM16415.1 | paenibacterin\_ABC-transporter\_D | BGC0000400 | Paenibacterin\_biosynthetic\_gene\_cluster | 24 | 30.7224848076 | 71 | 7.6e-12 |
| KIH99790.1 | hypothetical\_protein | BGC0001176 | Streptomonomicin\_biosynthetic\_gene\_cluster | 24 | 34.7738014855 | 70 | 2.2e-11 |
| AAK81823.1 | ABC\_transporter | BGC0000326 | Complestatin\_biosynthetic\_gene\_cluster | 22 | 30.0472653612 | 70 | 2.2e-11 |
| ACP43452.1 | McnB | BGC0000590 | Microcin\_N\_biosynthetic\_gene\_cluster | 23 | 36.326806212 | 66 | 3.2e-10 |
| ALE27499.1 | ABC\_transporter | BGC0001292 | A33853\_biosynthetic\_gene\_cluster | 23 | 21.471978393 | 66 | 3.2e-10 |
| AAP03990.1 | putative\_microcin\_L\_transport\_protein | BGC0000588 | Microcin\_L\_biosynthetic\_gene\_cluster | 23 | 32.4105334234 | 65 | 7.2e-10 |
| CAE55703.1 | probable\_microcin\_H47\_secretion\_ATP-binding\_protein\_mchF | BGC0000589 | Microcin\_M\_biosynthetic\_gene\_cluster | 22 | 32.478055368 | 64 | 1.2e-09 |
| BAL72550.1 | lariatin\_biosynthetic\_protein | BGC0000575 | Lariatin\_biosynthetic\_gene\_cluster | 23 | 32.2754895341 | 64 | 1.6e-09 |
| CAJ44959.1 | MchF\_protein | BGC0000587 | Microcin\_H47\_biosynthetic\_gene\_cluster | 22 | 32.478055368 | 62 | 3.6e-09 |
| AAL08400.1 | MceG | BGC0000586 | Microcin\_E492\_biosynthetic\_gene\_cluster | 22 | 32.478055368 | 62 | 3.6e-09 |
| BAI23327.1 | putative\_ABC-transporter | BGC0000949 | A-500359s\_biosynthetic\_gene\_cluster | 22 | 34.9763673194 | 62 | 6.1e-09 |
| AEG64672.1 | ABC\_transporter-like\_protein | BGC0000379 | Laspartomycin\_biosynthetic\_gene\_cluster | 24 | 27.6164753545 | 59 | 3e-08 |
| CAA90021.1 | PepT | BGC0000543 | Pep5\_biosynthetic\_gene\_cluster | 21 | 33.8284942606 | 56 | 4.3e-07 |
| CAM02316.1 | ABC\_transporter\_protein,\_ATP-binding\_component | BGC0000349 | Erythrochelin\_biosynthetic\_gene\_cluster | 21 | 28.5617825793 | 54 | 1.6e-06 |
| ABI30230.1 | McdT | BGC0000526 | Macedocin\_biosynthetic\_gene\_cluster | 22 | 33.2883187036 | 53 | 2.8e-06 |
